# Flexible nanoporous tunable electrical double layer biosensors for sweat diagnostics

**DOI:** 10.1038/srep14586

**Published:** 2015-09-30

**Authors:** Rujuta D. Munje, Sriram Muthukumar, Anjan Panneer Selvam, Shalini Prasad

**Affiliations:** 1 Department of Bioengineering, University of Texas at Dallas, 800 W. Campbell Road, EC 39, Richardson, TX 75080; 2 Enlisense LLC, 1813 Audubon Pond Way, Allen, TX 75013.

## Abstract

An ultra-sensitive and highly specific electrical double layer (EDL) modulated biosensor, using nanoporous flexible substrates for wearable diagnostics is demonstrated with the detection of the stress biomarker cortisol in synthetic and human sweat. Zinc oxide thin film was used as active region in contact with the liquid i.e. synthetic and human sweat containing the biomolecules. Cortisol detection in sweat was accomplished by measuring and quantifying impedance changes due to modulation of the double layer capacitance within the electrical double layer through the application of a low orthogonally directed alternating current (AC) electric field. The EDL formed at the liquid-semiconductor interface was amplified in the presence of the nanoporous flexible substrate allowing for measuring the changes in the alternating current impedance signal due to the antibody-hormone interactions at diagnostically relevant concentrations. High sensitivity of detection of 1 pg/mL or 2.75 pmol cortisol in synthetic sweat and 1 ng/mL in human sweat is demonstrated with these novel biosensors. Specificity in synthetic sweat was demonstrated using a cytokine IL-1β. Cortisol detection in human sweat was demonstrated over a concentration range from 10–200 ng/mL.

Wearable health-monitoring devices represent an exciting opportunity in healthcare as it promises to be the disruptive technology changing the status quo in patient care. Providing patients with tools to track their own conditions, they could be empowered to take responsibility for their own health. Sweat contains valuable medical information and is most suitable body fluid for designing non-invasive diagnostics platforms for consumer healthcare applications as compared to blood or urine. Sweat can be used to monitor basic metabolic panel ion levels such as sodium, potassium and chloride^1^. It can be used to monitor pathophysiology of hypoxia, ischemia through lactic acid etc^2^. Cortisol, a generic biomarker for stress is expressed in sweat with a wide dynamic concentration range which can be utilized to monitor physical changes such as fatigue during exercise as well as during military training and missions and is also of relevance as a biomarker in detection of a number of health conditions such as cystic fibrosis^3^. Varying levels of cortisol are indicators of stress disorders such as Cushing’s syndrome and Addison’s disease^3^. Researchers have been trying to acquire this information from sweat using various lab-on a-chip and label free bio-electronic platforms towards designing body-worn monitoring devices. We used cortisol in this study to demonstrate the capability of the flexible bio-electronic platform for sweat based diagnostic and related applications for wearable electronics. The challenge in designing wearable biosensing devices for biomarker diagnostics lies in the ability to detect and quantify the target molecules from very small volumes of perspired sweat during normal periods of activity. In this study we demonstrate electrical double layer biosensors on flexible substrates that utilize 3–5 μL sample volume of synthetic and human sweat for ultra-sensitive and selective biomolecule detection.

Electric field control of charge carrier density in a semiconductor has been identified as a significant method for tuning the electronic states of condensed matter. When a semiconducting material interacts with liquid electrolytes, an electrical double layer (EDL) is formed, which can have significantly high charge carrying capacity , at time as high as 8.0 × 10^14^ cm^−2^ at ionic liquid/solid interfaces^4^. The EDL formed at semiconductor/liquid interfaces is majorly capacitive, due to the presence of a large interfacial capacitance and the ability for high-density charge accumulation and hence can be electrically tuned to achieve desired mode of operation. The double layer capacitance (C_EDL_) however is typically enhanced at the liquid-semiconductor interfaces when the interface is used for biosensing as result of formation of biomolecular complexes, especially in the case of affinity based electrical immunoassays^5^. C_EDL_ changes actively with the concentration of biomolecular binding. The change in this C_EDL_ can be transduced with the required amplification by electrically modulating the high density charge carriers at the semiconductor-electrolyte interface. C_EDL_ can then be used for quantitating biosensor device response to biomolecular interactions. The ability to enhance the charge storage within the EDL can be used to obtain enhanced sensitivity and wider dynamic range in detecting the target species due to enhancement in the signal to noise ratio.

One of the ways to enhance charge storage and signal transduction from a non-Faradaic C_EDL_ is through nanoconfinement^6^, which refers to the physical mechanism of confining molecules in size matched spaces. This is mainly because nanoconfinement affects the electron transfer kinetics and enhances charge screening effects due to the electric field distribution inside the nanoporous structure^7^. The formation of EDL on the electrode surface by attracting counterions in solution occurs for screening the charges due to the mobile carriers in the electrode surface. Thus if a molecule is more than a debye length away, it would not have any effect on the mobile charges in the electrode defying the purpose of label-free biosensing and electrostatic tuning of semiconductor surface^8^. For planar substrates used for biosensing the charge screening has to be adjusted by controlling the ionicity of buffer solution or by decreasing the length of capture probe molecules for example through the use of aptamers and peptide nucleic acids. In this study we are enhancing the charge screening through the approach of nanoconfinement using nanoporous substrate. More importantly it also results in amplified C_EDL_ that facilitates measurable capacitance values for characterizing the changes at the liquid/solid interfaces. It also helps in controlled size-based diffusion of molecules within a porous media. Thus the decreased pore size facilitates customized variations in EDL thickness based on different biomolecules. Nanotextured surface has been reported to be very effective in increasing the accessibility for effective binding of biomolecules to obtain ultra-sensitivity^9^. Due to the low C_EDL_ on planar substrates, several studies using three terminal devices reported to date calibrate biosensing performance through direct current (DC) measurements that utilize change in resistance instead of alternating current (AC) measurements that utilize change in impedance and/or capacitance at the liquid/solid interface^10^,^11^,^12^,^13^,^14^,^15^. In this study, we attempt to characterize biosensing performance using AC based measurement methods with the C_EDL_ amplified using nanoconfinement and contrast the output with DC based measurement methods. The performance and attributes of the novel biosensor can be successfully used in designing devices in direct contact with sweat for applications in wearable electronics operating at low voltages.

We used zinc oxide (ZnO) as the active region in a three electrode setup for biosensing as it is a biocompatible semiconductor material with a wide direct band gap of 3.37 eV and high isoelectric point (IEP) of ~9.5 along with tunable electrical properties. ZnO matrix is positively charged at pH 7. A higher band gap allows for higher breakdown voltages and ability to sustain larger electric fields. The superior IEP allows for enhanced adsorption of proteins due to higher electrostatic attraction^16^. Moreover, ZnO being a polar semiconductor can also address the issues of specificity in the biosensor. The surface terminations of ZnO can be used to achieve favorable linker chemistry. Particular deposition conditions can be used to achieve n-type ZnO material that grows in wurtzite crystal form. The advantage of this type of crystal form in ZnO is the availability of alternate stacked layers with zinc and oxygen terminations. These terminations have proved effective for the functionalization of linker molecules such as Dithiobis [Succinimidyl Propionate] (DSP) and (3-Aminopropyl) triethoxysilane (APTES) to build a bio-immunoassay and have higher sensitivity to adsorbed molecules^17^,^18^. Ability to modulate the surface states can be useful in achieving heightened specificity of the sensor.

We used Pulsed Laser Deposition (PLD) technique to deposit ZnO thin films in the active regions. PLD deposited thin films highly crystalline and uniform with stable electrical properties even after deposition on flexible substrates^19^. PLD deposited ZnO has also been used for glucose and cortisol detection, using enzyme based assays^20^,^21^. Figure 1 show ZnO deposited on nanoporous polyamide substrate and the schematic of the sensor device. Parylene is deposited and patterned on the nanoporous polyamide substrate to isolates the entire sensor surface except the ZnO channel area from the liquid. Parylene fabrication is followed by transverse electrode fabrication. ZnO is deposited and patterned on the nanoporous polyamide substrate within the area enclosed by Parylene so that there is small overlap with the transverse electrodes. The orthogonal, third electrode is then fabricated above ZnO region. Electrical isolation of the electrodes is verified post sensor fabrication to ensure functionality prior to biosensor characterization.

## Results

The spatial and temporal changes to the charges in the EDL were monitored using DC-transconductance measurements and AC-electrochemical impedance spectroscopy (EIS) measurements.

### Comparison of C_EDL_ obtained using planar glass substrate with nanoporous flexible substrate

In Fig. 2a the semiconductor-solution interface is formed when 3–5 μL of synthetic sweat is wicked on to the flexible biosensor. A potential of 10 mV is applied across transverse electrodes which results in the migration of the charged ions (sodium, potassium, chloride, ammonium, bicarbonate, phosphate and sulphate, etc.) present in synthetic sweat (pH ~ 4–8 and conductivity of solution is 14.2 mS/cm) to form the EDL. When the dispensed solution comes in contact with the semiconductor surface the EDL is modulated through the accumulation or conjugation of biomolecules at the liquid/semiconductor surface. EIS measurements with voltage frequency ranging from 1 Hz to 1 kHz are performed to validate the enhanced charge accumulation in the EDL. C_EDL_ was derived from Z_imag_, the imaginary part of the impedance and based on the equivalent circuit model (see Supplementary Fig. S3 online). The bio-molecular interaction within EDL causes high charge carrier density accumulation in the semiconductor and as a result the capacitance of double layer increased from a baseline measurement of 2.93 μF·cm^−2^ to 3.53 μF·cm^−2^ and 4.95 μF·cm^−2^ for the detection of cortisol at its lowest and highest concentration from 1 pg/mL to 100 ng/mL respectively. In order to verify that the nanoconfinement enables high EDL capacitance, EIS performance is compared for sensors fabricated with ZnO on planar glass substrate and with ZnO on nanoporous flexible substrate.

Figure 2b shows the comparison of C_EDL_ obtained from ZnO thin films after functionalizing it with DSP linker on ZnO on planar glass substrate and ZnO on nanoporous polyamide substrates (directing reader to supplementary online). At this immunoassay stage of the sensing surface functionalized with the linker, the nanoporous polyamide substrate produced capacitance of 1.36 μF·cm^−2^ to 0.13 μF·cm^−2^ from 1 Hz to 1 kHz frequency. In the case of glass substrate the value of C_EDL_ was observed to be one order or more lower from 0.045 μF·cm^−2^ to 0.0045 μF·cm^−2^ from 1 Hz to 1 kHz frequency. The C_EDL_ obtained from ZnO thin films for each of the immunoassay steps on nanoporous polyamide as well as planar glass substrates is compared in Fig. 2c. Nanoporous polyamide substrate produced a baseline capacitance of 4.42 μF·cm^−2^ to 1.22 μF·cm^−2^ from synthetic sweat in the absence of cortisol from 1 Hz to 1 kHz frequency and it varied from 4.72 μF·cm^−2^ to 1.18 μF·cm^−2^ for 1 pg/mL cortisol and from 7.6 μF·cm^−2^ to 1.58 μF·cm^−2^ for 100 ng/mL cortisol concentrations over the same frequency range. In case of glass substrate, the value of C_EDL_ was observed to be at least one order lower from 0.23 μF·cm^−2^ to 0.076 μF·cm^−2^ from synthetic sweat in the absence of cortisol (baseline) from 1 Hz to 1 kHz frequency and it varied from 0.28 μF·cm^−2^ to 0.07 μF·cm^−2^ for 1 pg/mL cortisol and from 0.44 μF·cm^−2^ to 0.14 μF·cm^−2^ for 100 ng/mL cortisol concentration over the same frequency range. For planar electrodes the applied potential to the EDL needs to be substantially high i.e. in the range of 1–10 V to be able to achieve similar capacitance modulation as observed for nanoporous substrate. But application of potential in higher range can cause damage to the biomolecules such as proteins, causing them to denature. This issue is not seen on the nanoporous flexible substrate due to the enhanced charge accumulation resulting from nanoconfinement at lower voltages in the range of 10–50 mV.

### Calibration of sensor using AC modulation in synthetic sweat

The modulation in C_EDL_ was captured using AC measurements in the form of EIS. Figure 3 shows the results of calibration of EIS measurements for cortisol concentrations of 1 pg/mL to 100 ng/mL along with the measurement at zero dose, which is considered to be baseline measurement. This baseline measurement was carried out for the synthetic sweat buffer of pH ~ 4–8, on nanoporous flexible substrate with ZnO. It was observed that the baseline measurement (in terms of Z_mod_) at 1 Hz varied from 312 kΩ to 340 kΩ and at 1 KHz from 1.6 kΩ to 2.4 kΩ within the pH range of 4–8 (see Supplementary Fig. S1 online) and thus did not vary substantially. Further measurements were carried out using synthetic sweat at pH 8. EIS measurements track the dynamics of changing Z_real_ i.e. resistive and Z_imag_ i.e. imaginary (mainly capacitive) impedance of the sensor system. Hence EIS can be used to find out critical performance parameters of sensors such as limit of blank (LOB), limit of detection (LOD) and linear dynamic range (LDR).

The performance of sensors on planar vs. nanoporous substrate for cortisol detection was compared based on these parameters (refer to the Supplementary online). Five sensors were tested for their performance with EIS using cortisol. The dynamic changes in Z_imag_ and Z_mod_ (total impedance) for ZnO deposited nanoporous flexible substrate are represented in Fig. 3a,c respectively. It can be observed from Fig. 3a,c that the baseline when Z_imag_ variations are observed is 340 kΩ and that of Z_mod_ variations is 323 kΩ and does not differ substantially. Thus Z_real_ (real impedance due to resistive components such as buffer solution) part of total impedance does not affect the measurements and both Z_mod_ and Z_imag_ can be used to observe impedance variations. Figure 3b represents the changes in Z_imag_ for planar glass substrate. The variation in Z_imag_ for nanoporous substrate in Fig. 3a was observed to be from 260 kΩ to 165 kΩ and was found to be 5100 kΩ to 3260 kΩ for planar substrate as shown in Fig. 3b as the cortisol concentration is varied from 1 pg/mL to 100 ng/mL. Lower Z_imag_ in case of nanoporous substrate indicates higher C_EDL_ value as the Z_imag_ values are measured at same frequency for both substrates. These results demonstrate that there is higher biomolecular binding causing enhanced charge accumulation in case of nanoporous flexible substrate. Figure 3d compares critical performance parameters of sensors built on two substrates i.e. planar glass and nanoporous flexible polyamide substrate. The LOB, LOD and LDR were calculated from Fig. 3a,b in terms of capacitance at the interface. The specific signal threshold (SST) was calculated keeping in mind a signal to noise ratio (SNR) of minimum 3 (refer to the Supplementary online). The LOD and LDR for planar glass were found to be at 10 pg/mL and 0.13 μF·cm^−2^ respectively whereas for nanoporous polyamide substrate displayed an LOD and LDR of 1 pg/mL and 2.9 μF·cm^−2^ respectively. The percentage ratio of LDR to LOB was 45% for planar glass whereas polyamide substrate showed the ratio to be 65%. The dynamic range is in terms of capacitance variation was observed to be higher by at least one order for polyamide substrate as shown in Fig. 3d. The percentage change in impedance shown in the inset of Fig. 3a–c is calculated by finding the relative variation of Z_imag_ as well as Z_mod_ impedance from the baseline measurement using expression ((Z_baseline_- Z)*100/ Z_baseline_). The percentage of Z_imag_ impedance change is observed to be increasing from 19.6% to 48.6% for the range of 1 pg/mL to 100 ng/mL cortisol for polyamide substrate and for planar glass was found to be varying from 2.5% to 37.9%, where 2.5% for 1 pg/mL is not beyond SST. Since the charge carrier density at the interface is higher in case of polyamide substrate, it is reflected in the total capacitance at the interface for two substrates at each immunoassay step. This data confirms the wider dynamic range for polyamide substrate as compared to planar glass substrate sensors when tested with EIS.

### Calibration of sensor using DC modulation in synthetic sweat

DC measurements included measuring standard transfer characteristics of three electrode sensor system. Changes in transverse current were observed by varying orthogonal and transverse voltages, one at a time while the other voltage was kept constant. Five sensors fabricated separately on nanoporous substrates were tested for the consistency in performance using cortisol. Figure 4a shows change in transverse current as orthogonal voltage is varied from −1 V to 1 V for different bio-immunoassay steps. These measurements are taken at a constant transverse voltage of 0.1 V. The variation of current from 4.1 mA to 2.5 mA due to different cortisol concentrations of 1 pg/ml to 100 ng/mL is observed. It is evident from Fig. 4a that baseline was differing by less than 0.5 mA over the range of 1 pg/mL and 100 pg/mL cortisol concentrations. The higher concentrations showed less than 2 mA deviation from the baseline current response. Figure 4b shows the transverse current vs. transverse voltage characteristic for different steps of the bio-immunoassay. These measurements were taken at a constant orthogonal voltage of 0.1 V. The Fig. 4d shows the slopes of the transfer characteristic calculated at each immunoassay step (ΔI/ΔV) in transverse direction. This indicates change in conductance between two electrodes as different biomolecules are functionalized on ZnO surface. It was observed that the slopes of the graphs varied from 19.1 to 8.7. The slopes at 1 pg/mL and 100 pg/mL concentration were 12.8 and 12.7 respectively very close to the slope of baseline measurement of 13. The antibody-hormone EDL formation is not being detected by DC measurement as the DC voltage applied is at a constant frequency and therefore is unable to read into the charge modulation occurring due to bioconjugation in EDL. The range of change in slope in Fig. 4d for 1 pg/mL to 100 ng/mL was 12.8 (2%) to 8.7 (33%) wrt baseline, where the baseline measurement was almost overlapping with output curves of lower concentrations 1 pg/mL and 100 pg/mL. The specific signal threshold was at 9.7 (25.4%) and the limit of detection was at 100 ng/mL.

This analysis establishes the limitation of transverse DC measurements in detection of lower concentrations of analytes. Figure 4b inset shows the output curves when transverse current measurements were carried out by varying the transverse voltage from 0 V to 3 V and vice versa. Hysteresis was seen for higher transverse voltage range beyond 1.5 V which indicates of the possibility of biomolecules denaturing at higher applied potentials. From the Fig. 4, it is confirmed that DC measurements are inadequate for obtaining a wider dynamic range of detection.

### Comparison of senor performance using AC and DC modulation

In order to assess whether AC or DC modulation is most suitable for measuring biosensor performance, capacitance was extracted from both the output curves. For AC (EIS) measurements, capacitance (C_EDL_) is derived from Z_imag_ part of the impedance measured.

The slope of the transfer characteristic where orthogonal voltage is varied can be termed as transconductance (g_m_). This value can be used to derive the interfacial capacitance (C_in_) observed at the ZnO-solution interface by using following equation (1).
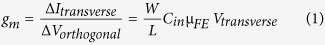
where, W and L are width and length of the ZnO thin film and μ_FE_ is the mobility of ZnO charge carriers^4^. The comparison of C_EDL_ and C_in_ is represented in Fig. 5a. These parameters are derived for every immunoassay step. It can be seen that C_EDL_ values range between 4.42 μF·cm^−2^ to 7.6 μF·cm^−2^ for zero dose cortisol concentration to 100 ng/mL concentration whereas the C_in_ quantity varies from 0.97 μF·cm^−2^ to 0.02 μF·cm^−2^. The capacitance extracted for the ZnO-solution interface captured using DC modulation (C_in_) is at least one order lower than capacitance obtained from AC modulation (C_EDL_) for the same concentration of cortisol dispensed at the interface implying the C_EDL_ belongs to the imaginary component of the impedance. This proves that AC modulation is more sensitive as a transduction mechanism for the charge modulation at the liquid/semiconductor interface than DC modulation. It can be concluded that it is more effective to calibrate the instrument using AC measurements.

The specificity of the sensor is tested by using a non-specific cytokine IL-1β found in sweat. The results of percentage change in resistance for AC modulation and percentage change in impedance for DC modulation due to interaction of IL-1β molecules with α-cortisol are shown in Fig. 5b,c. These output curves are compared with the corresponding calibration carried out using cortisol with α-cortisol. The EIS modulation using cortisol molecule over 1 pg/mL to 100 ng/mL showed percentage change in impedance from 19.6% to 48.6% whereas for IL-1β antigen it was 2.4% to 4.2%. Similarly for DC modulation, percentage change in resistance when tested with cortisol was found to be 2.8% to 37.3% with signal threshold at 15% whereas for IL-1β it was found to be 0.1% to 6%. This indicates that IL-1β antigen when used with α-cortisol and tested with DC and EIS modulation does not display heavy antibody-antigen interaction due to signal below noise level. This establishes that the biosensor is being able to distinguish between specific vs. non-specific target analytes and that EIS modulation shows a wider dynamic range in terms of change in impedance as compared to dynamic range seen in DC modulation.

### Calibration of sensor using AC modulation in human sweat

We performed dose calibration experiments using human sweat for cortisol concentration of 1 ng/mL to 200 ng/mL. Since human sweat is a complex medium, we used higher concentration of 500 μg/mL of cortisol antibody (see Supplementary Fig. S2 online). The results shown in Fig. 6 are based on the Z_imag_ measurements obtained at 100 Hz frequency for varying cortisol concentration in human sweat as we observed the highest SNR (signal-to-noise ratio) at this frequency. It can be observed from the inset of Fig. 6a that the percent change in impedance over the cortisol concentration in human sweat of 1 ng/mL to 200 ng/mL varies from 10% to 42%. The specific signal threshold is beyond 1 ng/mL concentration. Thus the limit of detection in human sweat is found to be at 1 ng/mL. However, the change in slope between 1 ng/mL to 10 ng/mL is not substantial. Thus we achieve LDR from 10 ng/mL to 200 ng/mL. As per the literature, cortisol concentration range in human sweat is reported ~8 ng/mL to 140 ng/mL^3^. The fractional change in dose Z_imag_ impedance measurements with respect to the baseline Z_imag_ impedance measurements for varying cortisol doses in human sweat is shown in Fig. 6b. This response was fitted using exponential fit and the correlation was found to be 0.9961. Figure 6b also shows the response for two test samples (human sweat with 50 ng/mL and 150 ng/mL cortisol dose concentrations respectively). The table inset in Fig. 6b compares the actual concentrations of cortisol tested to the measured values of cortisol concentration estimated from exponential fit. The measured values of cortisol estimated from the calibration dose response varied from the actual value of cortisol by an average of 12.5%, which is within the permissible limits of clinical standards as published by the Clinical And Laboratory Standards Institute^22^. Observed correlation between the measured and expected values of the test samples demonstrate analytical precision of the sensor. It can be concluded that the sensor performance is effective in clinically relevant range and the flexible sensor has demonstrated the potential for practical diagnostic applications.

## Discussion

Wearable diagnostics biosensors need to demonstrate enhanced sensitivity, with detection of the target species in clinically relevant target regimes from low sample volumes (<10 μL ranges). Additionally, they need to operate at very low bias potential enabling low power operation and consumption. In this paper we have presented a flexible three electrode biosensor on nanoporous polyamide substrates for detection and quantification of cortisol; a physiological stress marker that is expressed in sweat in its clinically relevant regime from 8 ng/mL to the 140 ng/mL^3^. The findings from this research are that in order to achieve sensitivity as well as a linear dynamic range within the concentration regime using ultra low volumes (<5 μL; corresponding to ambient sweat) and an electrical detection method, AC voltage based measurement of the EDL impedance is more suitable than direct current measurement of the interfacial capacitance and transconductance characteristics. The results of Figs 2 and 3 demonstrate the significance of AC-electrochemical impedance spectroscopy (EIS) measurements and its impact on biosensor performance in synthetic sweat. The highest percentage change of 48.6% is obtained for the highest concentration of cortisol at 100 ng/mL and limit of detection (LOD) of 1 pg/mL is achieved in nanoporous substrates. The utility of the nanoporous substrate has been further explored with cortisol detection in human sweat. Figure 6 demonstrate the impact of AC-electrochemical impedance spectroscopy (EIS) measurements on nanoporous substrate biosensor performance in human sweat. The nanoporous substrate acts like size exclusion sieve thus enabling the targeted binding of biomolecules even at very low concentrations. Therefore, the linear dynamic range is still preserved from 10 ng/mL to 200 ng/mL while the limit of detection (LOD) was found to be at 1 ng/mL. The higher LOD in human sweat was due to the interferents present in the sweat matrix. The confinement of targeted biomolecules in the nanoporous substrate causes high charge accumulation in the double layer resulting across a ZnO–solution interface. Increased C_EDL_ range will allow us to scan the EDL more distinctly enabling the clear discrimination of smallest dynamic changes occurring due to biomolecule binding. Hence, this capability is utilized to obtain a stable signal from human sweat. Additionally due to the linear dynamic range over the cortisol concentration regime in the ng/mL, it is possible to estimate cortisol concentrations at diagnostically relevant values with variances within the acceptable limits of clinical laboratory standards. Higher charge accumulation with increasing concentration of targeted biomolecules is very advantageous for operating the sensor at lower voltages due to amplified output. This enabled us in achieving high percent change in impedance of 10% at 1 ng/mL of cortisol and 42% for 200 ng/mL of cortisol , with a LDR of 30% when the sensor was tested in a complex medium like human sweat.

Dispensing different molecules in liquid near metal oxide surface is equivalent to applying different electric potentials, causing electrostatic gating effect which in turn changes the carrier density. But resistive changes derived from DC measurements are inadequate to detect these variations for smaller cortisol concentrations as concluded from Fig. 4. DC measurements primarily captured the resistive changes in the ZnO-liquid interfaces primarily due to either change in mobility of carriers in ZnO or change in the density of carriers in ZnO^13^. It is established from Fig. 4a,b that the transverse current does not vary substantially (~2% change in resistance) with increasing orthogonal voltage. This proves that the cortisol binding over a concentration range of 1 pg/mL to100 ng/mL does not cause substantial changes in carrier mobility in the semiconducting ZnO. Hence the resistive changes observed with different assay steps must be due to changes in charge carrier density at the interface on the ZnO semiconductor when it comes in contact with the liquid. Since there is a substantial change in charge carrier density at the interface, it is well reflected in the capacitance measured across the interface. Hence the sensor performance is lowered when limit of detection is calculated based on resistive changes in terms of slope variation and not the capacitive variations as cortisol concentration is varied.

C_in_ interfacial capacitance derived using transconductance of DC characteristics is considerably low compared to the C_EDL_ obtained from EIS measurements as shown in Fig. 5a. As per equation 1, the C_in_ depends on mobility of charge carriers, dimension of the biofunctionalized area over ZnO surface, transconductance and the applied transverse bias. As discussed above, the mobility of charge carriers is not affected due to dispensing of particular cortisol concentration as the transconductance does not change substantially. Thus the C_in_ can only be improved by either decreasing the W/L ratio i.e. by shrinking the dimensions of the bio-functionalized semiconductor area in the sensor or by increasing the applied transverse potential. Reduction in area has the negative effect in reducing the number of biomolecules available for sensing. Increasing potential has the negative effect of higher power consumption for wearable device applications. The demonstrated DC measurements were taken at ±1 V varying orthogonal voltage and 0.1 V transverse voltage which is two orders higher than the voltage applied for EIS which was 10 mV. The transverse DC potential bias applied cannot be increased beyond 1.5 V as the higher voltages applied for long duration might damage the biomolecules under test^23^,^24^. C-V measurements under DC potential can only be effective for biosensing devices if equivalent or higher ranges of capacitance C_in_ can be obtained as compared to C_EDL_ at lower applied voltages. Thus DC is not amenable for development of ultra-low power and low voltage applications, which is one of the major requirements for development of wearable sensors^25^. Although DC measurements would be simpler for assembly and integration of sensor device but it also comes with limitations such as higher DC voltages cause denaturation of biomolecules or capture probes under test. In conclusion, AC based electrochemical impedance spectroscopy (EIS) is an effective method for characterizing the biomolecular charge accumulation in the electrical double layer formed at the liquid/semiconductor i.e. ZnO in the current biosensor configuration using nanoporous flexible polyamide substrates for ultrasensitive detection of cortisol in synthetic and human sweat. This work is significant from the perspective of realizing ultra-low power wearable and high performance biosensors.

## Methods

### Materials

Polyamide substrates were ordered from GE Healthcare Life Sciences (Piscataway, NJ, USA) with 0.2 μm pore size. The linker molecule Dithiobis [Succinimidyl Propionate] (DSP) and its solvent Dimethyl Sulfoxide (DMSO) is ordered from Thermo Fisher Scientific Inc. (Waltham, MA, USA). The α-cortisol antibody and cortisol hormone (Hydrocortisone) was ordered from Abcam (Cambridge, MA, USA). IL-1β antigen was bought from Thermo Fisher Scientific Inc. (Waltham, MA, USA). The antibody was diluted in 1X phosphate buffered saline (PBS, Thermo Fisher Scientific Inc., Waltham, MA, USA). Both cortisol hormone and IL-1β are diluted using synthetic sweat. The synthetic sweat was prepared as per the recipe stated in Table 2 of M.T. Mathew *et al.*^26^. The pH range is varied by varying the concentration of the components. Human sweat was purchased from Lee biosolutions Inc. (St. Louis, MO, USA), where it was collected from single human donor with pH ~ 4–5. No preservatives have been added to this product and it was stored unfiltered at below −20 °C.

### Sensor Fabrication

**Figure 1:**
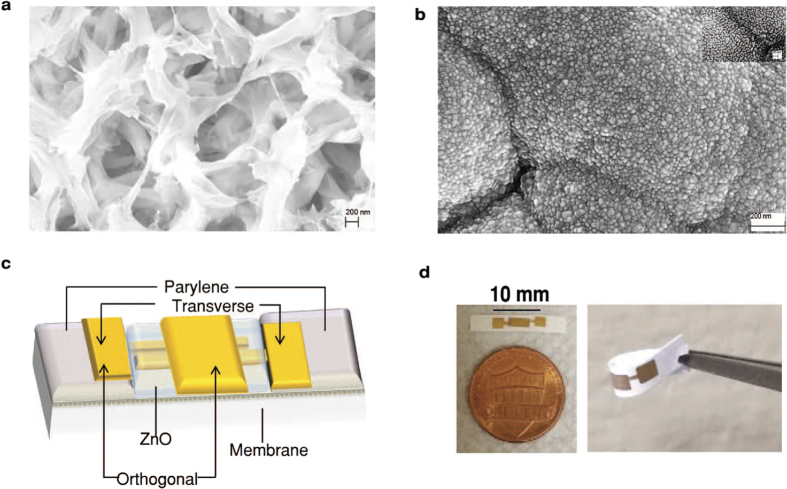
Structural characteristics of sensor. (**a**) SEM image of nanoporous polyamide substrate. (**b**) SEM image of bio-functionalized area of ZnO in sensor on nanoporous polyamide substrate. (**c**) Schematic of the biosensor configuration (**d**) actual sensor image.

**Figure 2:**
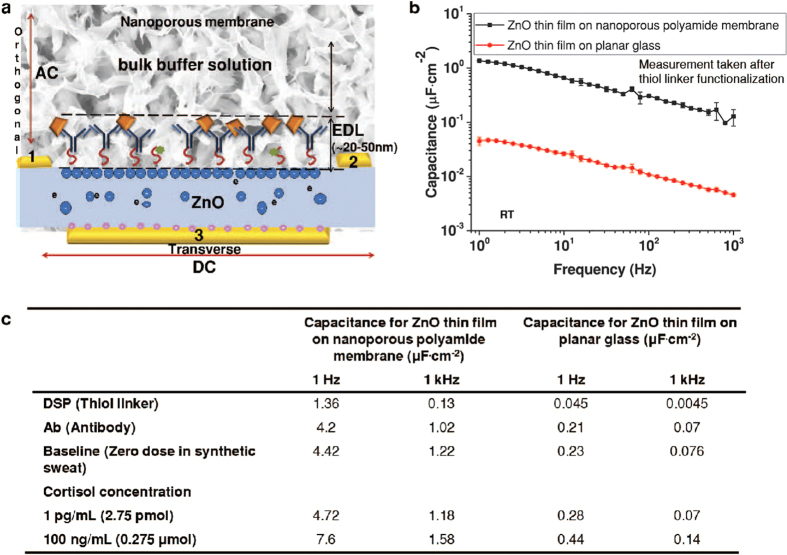
Comparison of the formation of EDL at the interface of the zinc oxide thin film on nanoporous flexible polyamide substrate and on planar glass substrate. (**a**) cross-section of ZnO surface on the flexible nanoporous polyamide substrate with modification to EDL during immobilization of biomolecules. (**b**) C_EDL_ measured at room temperature using impedance spectroscopy when ZnO surface is functionalized with DSP linker between glass substrate vs. nanoporous polyamide substrate. (**c**) Capacitance ranges for ZnO on flexible nanoporous polyamide vs. ZnO on planar glass substrates for each step of the assay over the frequency ranges associated with cortisol detection in synthetic sweat.

**Figure 3:**
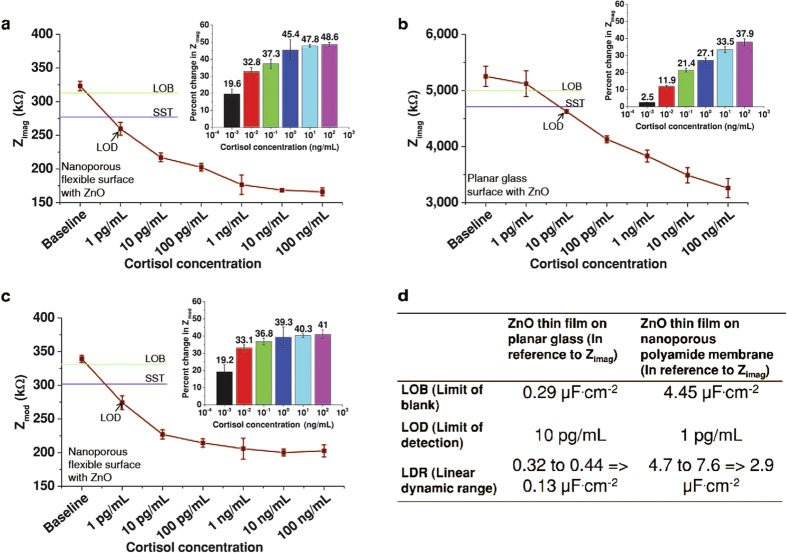
Sensor performance evaluation in synthetic sweat using AC modulation through EIS. (**a**) Change in imaginary impedance Z_imag_ for varying cortisol concentration of 1 pg/mL to 100 ng/mL on nanoporous polyamide surface with ZnO. (**b**) Change in imaginary impedance Z_imag_ for varying cortisol concentration of 1 pg/mL to 100 ng/mL on planar glass surface with ZnO. (**c**) Change in total impedance Z_mod_ for varying cortisol concentration of 1 pg/mL to 100 ng/mL. In all cases inset represents percentage change in impedance with respect to baseline i.e. zero dose. (**d**) Comparison of sensor performance parameters obtained after analyzing EIS measurements for ZnO thin films on planar glass vs. nanoporous flexible substrate.

**Figure 4:**
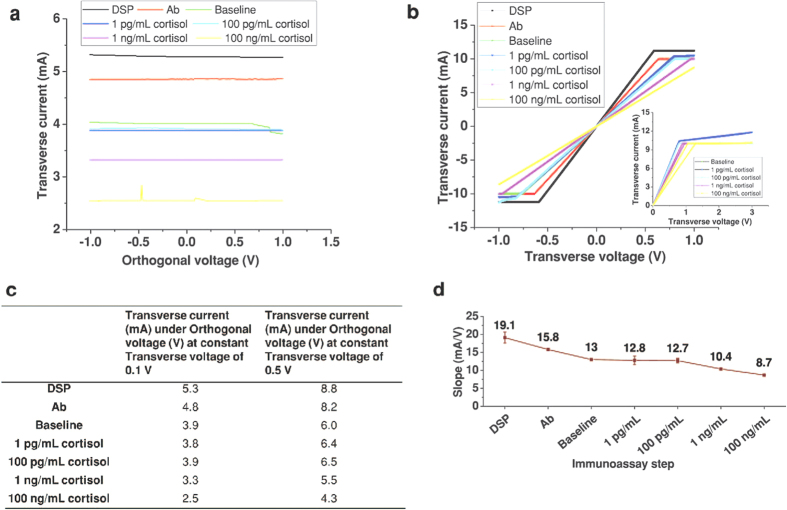
Sensor performance evaluation in synthetic sweat using DC modulation. (**a**) Change in transverse current for varying orthogonal voltage at constant transverse voltage of 0.1 V for each immunoassay. (**b**) Change in transverse current for varying transverse voltage at constant 0 V orthogonal voltage for each immunoassay step. Inset shows transverse current-voltage output curve with applied transverse voltage from 0 V to 3 V and 3 V to 0 V for each immunoassay step. (**c**) Transverse current values under orthogonal voltage for each step of immunoassay for constant transverse voltage at 0.1 V and 0.5 V. (**d**) Change in the slope for every immunoassay step calculated from Fig. 4b.

**Figure 5:**
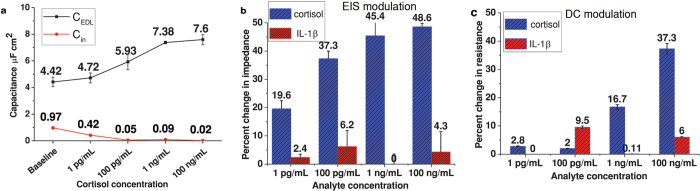
Sensor performance comparison between DC modulation and EIS modulation in synthetic sweat. (**a**) Variation of capacitance due to DC and AC measurement extracted for different cortisol concentrations from baseline to 100 ng/mL. (**b**) Cross-reactivity measurements with IL-1β and its comparison with cortisol detection in terms of percentage change using EIS modulation. (**c**) Cross-reactivity measurements with IL-1β and its comparison with cortisol detection in terms of percentage change using DC modulation.

**Figure 6:**
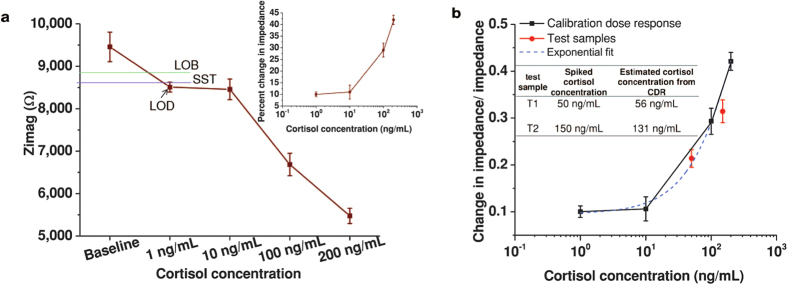
Sensor performance evaluation in human sweat using AC modulation through EIS. (**a**) Variation in imaginary impedance for cortisol concentration variation of 1 ng/mL to 200 ng/mL in human sweat. Inset shows the percent change in impedance. (**b**) Calibration dose response rationalization over the range of 1 ng/mL to 200 ng/mL. Inset shows test sample mapping using exponential fit.

Figure 1c shows the sensor platform deposited on nanoporous polyamide substrate substrate as well as the schematic of the stack of the materials. Parylene C of 1 μm thickness deposited using shadow mask prior to gold electrodes for insulation in Spin coating system (Specialty coating systems, Inc. Indianapolis, IN, USA). It is patterned such that it isolates the entire sensor surface except the ZnO channel area from the liquids. This will contribute in obtaining stable and reliable output response. Gold electrodes were patterned using shadow mask and deposited using e-beam cryo-evaporator. Pulsed laser deposition method was used to deposit ~90–100 nm ZnO thin films on flexible polyamide substrates at 10 mTorr pressure and 100 ^°^C temperature using PLD system (Neocera, LLC. Beltsville, Maryland, USA). ZnO is carefully patterned such that it overlaps with the two electrodes except at the contact pads. The third electrode made of gold is then patterned carefully above ZnO such that it does not short-circuit the other electrodes. Electrical isolation of the electrodes is verified post fabrication.

### Structural and electrical characteristics

Hall measurements were performed on ZnO deposited glass substrates using 8400 series HMS (Lake Shore Cryotronics, Inc.Carson, CA, USA). Hall measurements performed using glass substrate reveal the resistivity of the deposited film to be 0.02 Ω/cm and mobility was 18 cm^2^/V.s. In order to investigate the growth of ZnO on flexible porous polyamide substrate, structural characterization was done using SEM images captured using SUPRA SEM (Zeiss, Oberkochen,Germany). Figure 1a,b show SEM images captured at different stages of the sensor. The Fig. 1a is SEM of polyamide substrate and confirms the porous and fibrous entangled nature of polyamide substrate with a pore size of around 200 nm. This porous substrate will allow for easy flow of liquids to active layer of ZnO thus allowing for less incubation time. Figure 1b shows the ~20–30 nm diameter nanostructured ZnO on polyamide in the sensor focused in the channel area. Inset shows the zoomed image of the same clearly showing the ZnO exposed for molecular binding.

### Sensor performance

All the solutions are dispensed on the sensor from backside. ZnO surface was functionalized with 3–5 μL of 10 mmol DSP linker after incubation of 2 hours. The PBS wash was carried out followed by 15 min incubation of 3–5 μL of 10 μg/mL α-cortisol antibody. Various dilutions of cortisol in synthetic sweat from 1 pg/mL to 100 ng/mL were dispensed after antibody incubation. Each cortisol concentration was incubated for 15 minutes on the sensor surface prior to measurement. The specificity of the biochemical assay was tested by performing cross-reactivity experiments using the interfering protein molecule IL-1β antigen in synthetic sweat with cortisol antibody. Sensor calibration response was obtained in human sweat with increased cortisol concentration doses from 1 ng/ml to 200 ng/mL with n = 3 replicates of chips. The dependence of fractional change in dose impedance wrt baseline impedance on cortisol concentration was then rationalized. Two test sample chips with 50 ng/mL and 150 ng/mL cortisol concentration doses on each were also measured with 3 iterations. The average readings were then compared with the values obtained from calibration dose response in human sweat. These experiments were performed using both AC (EIS) and DC modulation on polyamide substrate at each step of building of the bio-immunoassay. EIS measurements were taken by recording current flow using a potentiostat (Gamry Instruments, Warminster, PA, USA) after applying 10 mV AC voltage with a frequency sweep of 1 Hz to 1 KHz. The sensor signal was recorded using a 4200 SCS (Keithley Instruments Inc. Cleveland, Ohio, USA) and a Summit probe station (Cascade Corporation, Portland, Oregon, USA) The orthogonal voltage of −1 V to 1 V was applied in steps of 0.01 V at constant 0.1 V transverse voltage. Also the current flow in transverse direction was measured by sweeping the transverse voltage from −1 V to 1 V in steps of 0.001 V at a constant orthogonal voltage of 0 V. All the measurements are carried out in dark and under ambient temperature conditions.

## Additional Information

**How to cite this article**: Munje, R. D. *et al.* Flexible nanoporous tunable electrical double layer biosensors for sweat diagnostics. *Sci. Rep.* **5**, 14586; doi: 10.1038/srep14586 (2015).

## Supplementary Material

Supplementary Information
