# Supplementary material for: Flexible nanoporous tunable electrical double layer biosensors for sweat diagnostics
Source: Sci Rep. 2015 Sep 30;5:14586. doi: 10.1038/srep14586 (PMC4588585; doi:10.1038/srep14586)
Supplement: Supplementary Information [file srep14586-s1.pdf]

# Flexible nanoporous tunable electrical double layer biosensors for sweat diagnostics

Rujuta D.Munje[1], Sriram Muthulumar[2], Anjan Panneer Selvam [1], Shalini Prasad[1]

[1]Department of Bioengineering, University of Texas at Dallas, 800 W. Campbell Road, EC 39, Richardson, TX 75080
[2] Enlisense LLC, 1813 Audubon Pond Way, Allen, TX 75013

**Supplementary Table S1. Dielectric constant of substrates used[1]**

| | **Dielectric constant (50 Hz to 1 KHz)** |
|---|---|
| **Polyamide** | 4-3 |
| **Parylene** | 3.15-3.10 |
| **Corning glass 7059** | 5-5.3 |

The materials are selected such that their dielectric constants do not vary significantly.

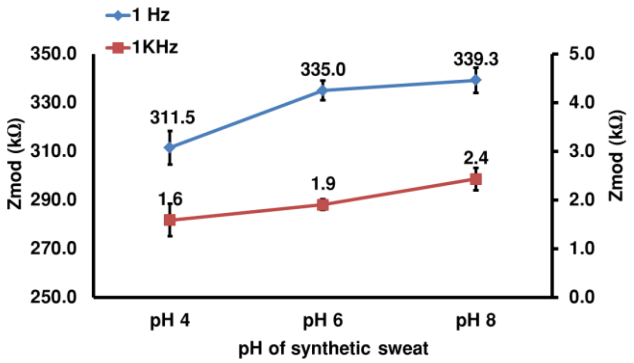


**Supplementary Figure S1 | Baseline (Zero dose) measurements at 1 Hz and 1 KHz by varying pH of synthetic sweat from 4 to 8**

The intent of Supplementary Fig. S1 is to demonstrate that at a particular frequency, value of total impedance does not vary significantly for different pH of synthetic sweat.

The $Z_{mod}$ value obtained at each frequency consists of $Z_{real}$ and $Z_{imag}$ components. The $Z_{imag}$ (mainly capacitive) component is frequency dependent and is inversely proportional to the frequency of measurement. Also the bulk of the electrode/electrolyte interface is scanned at higher frequencies which results into lowering of the resistive ( Zreal) component value. Thus the value of $Z_{mod}$ decreases as we increase the frequency. We chose the 1Hz to 1 kHz range of measurement as it was observed that at lower frequencies (< 1 kHz ) the $Z_{imag}$ (capacitive) component has higher contribution (~ 85% in this case) towards $Z_{mod}$ . Since the capacitive component $Z_{imag}$ represents the EDL capacitance, the perturbation to the EDL due to dispensing of different biomolecules will be more accurately captured at lower frequencies. At the frequencies lower than 1 Hz, the surface capacitance which behaves more like a constant phase element starts to contribute and hence we observe lowered contribution (~ 75%) of $Z_{imag}$. Thus we decided to observe the impedance changes in the range of 1 Hz to 1 kHz.

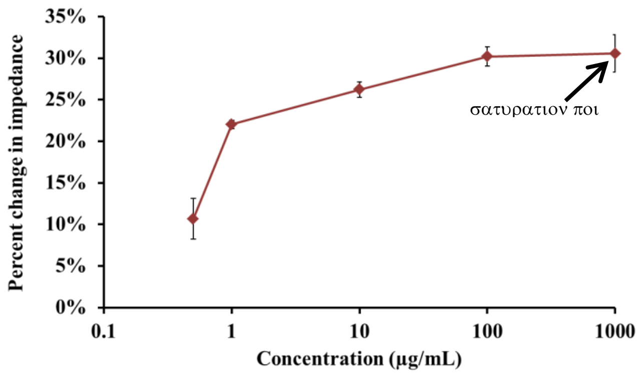


**Supplementary Figure S2 | Variation in percent change in imaginary impedance as the concentration of cortisol antibody is varied from 0.5 μg/mL to 1000 μg/mL.**

Antibody saturation study was performed to identify the minimum saturating dose of the antibody towards identifying the dose of the antibody that would be optimum for human sweat based cortisol dose response experiments. Human sweat is a complex medium. It was necessary to confirm the sufficient coverage of antibody on sensor surface while performing experiments with complex medium, human sweat. In the antibody saturation study we observed the change in $Z_{imag}$ (imaginary impedance) for varying cortisol antibody concentration of 0.5 μg/mL to 1000 μg/mL. It can be observed from Supplementary Fig. S2 that there was an inflection point at 1000 μg/mL concentration of the antibody, but the rate of change of slope was near zero when the sensor surface is tested with antibody between 100 μg/mL to 1000 μg/mL. Thus it was concluded to use 500 μg/mL of cortisol antibody concentration for experiments in human sweat.

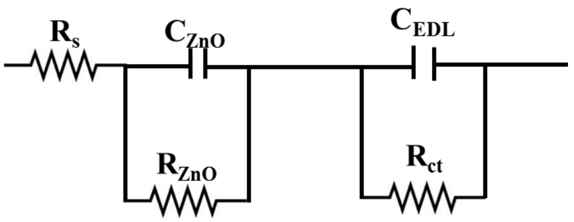


**Supplementary Figure S3 | Equivalent circuit diagram for the sensor system**

Equivalent circuit presented in Supplementary Fig. S3 can be interpreted for the given sensor system.

$R_s$: Solution resistance

$R_{ZnO}$: Resistance of ZnO thin film

$C_{ZnO}$: Capacitive effects of ZnO

$R_{ct}$: Charge transfer resistance

$C_{EDL}$: Double layer capacitance

This circuit has been previously established for ZnO based sensor studies[2].

**Definitions of the critical performance parameters.**

**LOB (Limit of Blank) :** The highest apparent analyte concentration expected to be found when replicates of a sample containing no analyte are tested[3]. LOB is estimated by measuring replicates of a blank sample and calculating the mean result and the standard deviation (SD)[3].

$\mathrm{LOB} = \mathrm{mean}_{\mathrm{blank}} + 1.645(\mathrm{SD}_{\mathrm{blank}})$……..Supplementary equation S(1)

**SST (Specific Signal Threshold)[4]:** The minimum impedance level interpreted, which is considered as signal level. A signal to noise ratio of 3 is decided and the specific signal threshold impedance as three times the noise signal is calculated. The noise level is defined as the difference in the reading between average antibody measurement and average baseline (zero dose) measurement.

**LOD (Limit of Detection):** The lowest analyte concentration likely to be reliably distinguished from the LOB and at which detection is feasible[3].

$\mathrm{LOD} = \mathrm{LOB} + 1.645(\mathrm{SD}_{\mathrm{lowest\ concentration\ sample}})$………..Supplementary equation S(2)

**REFERENCES:**


1 National Physical Laboratory. *Kaye & Laby Tables of Physical and Chemical constants*, <http://www.kayelaby.npl.co.uk/general_physics/2_6/2_6_5.html> (

2 Jacobs, M., Muthukumar, S., Selvam, A. P., Craven, J. E. & Prasad, S. Ultra-sensitive electrical immunoassay biosensors using nanotextured zinc oxide thin films on printed circuit board platforms. *Biosensors and Bioelectronics* **55**, 7-13 (2014).

3 Armbruster, D. A. & Pry, T. Limit of blank, limit of detection and limit of quantitation. *Clin Biochem Rev* **29**, S49-52 (2008).

4 McEnroe, R. J., Burritt, M. F. & Powers, D. M. *Interference testing in clinical chemistry; approved guideline*. (CLSI, 2005).
